# Supplementary material for: Tracking transcription factor mobility and interaction in Arabidopsis roots with fluorescence correlation spectroscopy
Source: eLife. 2016 Jun 11;5:e14770. doi: 10.7554/eLife.14770 (PMC4946880; doi:10.7554/eLife.14770)
Supplement: Supplementary file 3. — DOI: http://dx.doi.org/10.7554/eLife.14770.034 [file elife-14770-supp3.pdf]

Supplemental File 3. Equations, initial conditions, and parameter values used for simulations.

SHR-SCR Model in wild type conditions

$$\frac{dS_v}{dt} = k_1 - a_1 S_v + a_2 S_e - d_1 S_v$$
$$\frac{dS_e}{dt} = a_1 S_v - d_2 S_e$$
$$\frac{dS_{2e}}{dt} = k_2(C) S_e^2 - d_3 S_{2e}$$
$$\frac{dC}{dt} = k_3 \left(\frac{K_{1D}^2 C + K_{1D} SC + S_2 C}{K_{1D}^2 K_{2D} + K_{1D} K_{2D} S_e + K_{1D}^2 C + K_{2D} S_e + K_{1D} SC + S_2 C} \right) - d_4 C$$
$$\frac{dSC}{dt} = k_4 S_e C - d_5 SC$$
$$\frac{dS_2C}{dt} = k_5 S_{2e} C - d_6 S_2 C$$
$$[S_v(0), S_e(0), S_{2e}(0), C(0), SC(0), S_2C(0)] = (0.1, 0, 0, 0.1, 0, 0)$$

where $a_1 = \dfrac{D_1}{A_1}$, $a_2 = 0$, and $k_2(C) = \dfrac{L}{1 + e^{-k(C - C_0)}}$.

SHR-SCR Model in SCRi line

$$\frac{dS_v}{dt} = k_1 - a_1 S_v + a_2 S_e - d_1 S_v$$
$$\frac{dS_e}{dt} = a_1 S_v - a_2 S_e - d_2 S_e$$
$$\frac{dS_{2e}}{dt} = k_2(C) S_e^2 - d_3 S_{2e}$$
$$\frac{dC}{dt} = 0$$
$$\frac{dSC}{dt} = k_4 S_e C - d_5 SC$$
$$\frac{dS_2C}{dt} = k_5 S_{2e} C - d_6 S_2 C$$
$$[S_v(0), S_e(0), S_{2e}(0), C(0), SC(0), S_2C(0)] = (0.1, 0, 0, 300, 0, 0)$$

where $a_1 = \dfrac{D_1}{A_1}$, $a_2 = \dfrac{D_2}{A_2}$, and $k_2(C) = \dfrac{L}{1 + e^{-k(C - C_0)}}$.

Parameter	Biological meaning (units)	Initial Value	Estimated Value	Reference
k_1	Production of SHR in vasculature (Conc/hr)	300		Cruz-Ramirez et al, 2012
D_1	DC of SHR from vasculature to endodermis (μm^2/sec)	2.11		Experimentally determined
A_1	Area of vasculature cell (μm^2)	31.63		Experimentally determined
D_2	DC of SHR from endodermis to vasculature (μm^2/sec)	2.08		Experimentally determined
A_2	Area of endodermis cell (μm^2)	36.08		Experimentally determined
d_1	Degradation of SHR monomer in vasculature (1/hr)	1		Cruz-Ramirez et al, 2012
d_2	Degradation of SHR monomer in endodermis (1/hr)	1	1000	Estimated
L	Maximum value of k_2 (1/hr)	300	0.5	Estimated
k	Steepness of k_2 curve (1/Conc.)		0.1	Estimated
C_0	Midpoint of k_2 curve (Conc.)		360	Estimated
d_3	Degradation of SHR homodimer in endodermis (1/hr)	1		Cruz-Ramirez et al, 2012
k_3	Production of SCR (1/hr)	1100		Cruz-Ramirez et al, 2012
K_{1D}	Dissociation constant for SHR (Conc.)	1000		Cruz-Ramirez et al, 2012
K_{2D}	Dissociation constant for SCR (Conc.)	1000	500	Estimated
d_4	Degradation of SCR (1/hr)	1		Cruz-Ramirez et al, 2012
k_4	Formation of 1:1 SHR-SCR complex (1/hr)	5		Cruz-Ramirez et al, 2012
d_5	Degradation of 1:1 SHR-SCR complex (1/hr)	1		Cruz-Ramirez et al, 2012
k_5	Formation of 2:1 SHR-SCR complex (1/hr)	5		Based on k_4
d_6	Degradation of 2:1 SHR-SCR complex (1/hr)	1		Based on d_5

Parameter values. Note that all diffusion coefficients were converted to hours for estimation and simulation. Some parameters were estimated using experimental data: for the rest, the initial values were used in simulations. DC: diffusion coefficient
